# Unintended pregnancy is a risk factor for depressive symptoms among socio-economically disadvantaged women in rural Bangladesh

**DOI:** 10.1186/s12884-018-2097-2

**Published:** 2018-12-13

**Authors:** Pamela J. Surkan, Donna M. Strobino, Sucheta Mehra, Abu Ahmed Shamim, Mahbubur Rashid, Lee Shu-Fune Wu, Hasmot Ali, Barkat Ullah, Alain B. Labrique, Rolf D. W. Klemm, Keith P. West, Parul Christian

**Affiliations:** 1 Center for Human Nutrition, Department of International Health, Johns Hopkins Bloomberg School of Public Health, 615 North Wolfe St., Room E2519, Baltimore, MD 21205-2179 USA; 2 Department of Population, Family and Reproductive Health, Johns Hopkins Bloomberg School of Public Health, Baltimore, MD 21205-2179 USA; 3 The JiVitA Project, Johns Hopkins University in Bangladesh, Gaibandha, Bangladesh

**Keywords:** Unintended pregnancy, Pregnancy intention discordance, Perinatal depression, Depressive symptoms, Bangladesh

## Abstract

**Background:**

Little is known about the relation between unwanted pregnancy and intention discordance and maternal mental health in low-income countries. The study aim was to evaluate maternal and paternal pregnancy intentions (and intention discordance) in relation to perinatal depressive symptoms among rural Bangladeshi women.

**Methods:**

Data come from a population-based, community trial of married rural Bangladeshi women aged 13–44. We examined pregnancy intentions among couples and pregnancy-intention discordance, as reported by women at enrollment soon after pregnancy ascertainment, in relation to depressive symptoms in the third trimester of pregnancy (*N* = 14,629) and six months postpartum (*N* = 31,422). We calculated crude and adjusted risk ratios for prenatal and postnatal depressive symptoms by pregnancy intentions.

**Results:**

In multivariable analyses, women with unwanted pregnancies were at higher risk of prenatal (Adj. RR = 1.60, 95% CI: 1.37–1.87) and postnatal depressive symptoms (Adj. RR = 1.32, 95% CI: 1.21–1.44) than women with wanted pregnancies. Women who perceived their husbands did not want the pregnancy also were at higher risk for prenatal (Adj. RR = 1.42, 95% CI: 1.22–1.65) and postnatal depressive symptoms (Adj. RR = 1.30, 95% CI: 1.19–1.41). Both parents not wanting the pregnancy was associated with prenatal and postnatal depressive symptoms (Adj. RR = 1.34, 95% CI: 1.19–1.52; Adj. RR = 1.13, 95% CI: 1.06–1.21, respectively), compared to when both parents wanted it. Adjusting for socio-demographic and pregnancy intention variables simultaneously, maternal intentions and pregnancy discordance were significantly related to prenatal depressive symptoms, and perception of paternal pregnancy unwantedness and couple pregnancy discordance, with postnatal depressive symptoms.

**Conclusions:**

Maternal, paternal and discordant couple pregnancy intentions, as perceived by rural Bangladeshi women, are important risk factors for perinatal maternal depressive symptoms.

## Background

Unintended pregnancies are a major problem worldwide, with 40% of pregnancies estimated to be unintended in less developed regions [1, 2]. Unintended pregnancies refer to pregnancies not wanted at the time (mistimed) or at any time (unwanted) [3]. Econometric analyses indicate that contraception is cost-effective relative to the consequences of unintended pregnancies [4]. As such, family planning initiatives have attempted to curb unwanted pregnancies; however, major socio-economic disparities in access to contraception still exist in developing countries [5]. In Bangladesh, a substantial number of unwanted pregnancies are terminated legally through ‘menstrual regulation’ for which manual vacuum aspiration is used to safely establish non-pregnancy up to 8–10 weeks after a women’s missed period; yet, according to the 2007 Demographic and Health Survey about 30% of term live births are reported to be unintended [6].

A review of the literature suggested that, although findings are mixed, unwanted pregnancy is associated overall with adverse consequences, including limited antenatal care, short duration of breast feeding, adverse birth outcomes, and elevated infant and child mortality [7]. While there is a substantial literature on unintended pregnancy and mental health in developed countries, little is known about the relation between unwanted pregnancy and maternal mental health in lower- and middle- income countries [8]. Yet, high levels of common mental disorders, being predominantly maternal depressive symptoms, during pregnancy and postpartum (16 and 19%, respectively) have been estimated in lower- and middle-income countries [9]. In eastern Bangladesh, about one-third of women reported depressive symptoms between 34 and 35 weeks of pregnancy and 22% at 6–8 weeks postpartum [10, 11]. Furthermore, gendered inequalities related to reproductive decision-making and control exist within South Asia, with 48% of women in Bangladesh reporting that their husbands make decisions about their health and accessing healthcare services [12, 13]. Given this context, we were particularly interested in studying discordance of wantedness between couples in relation to depressive symptoms.

The principal aim of this study was to evaluate the relation between maternal and paternal pregnancy intentions and prenatal (in the 3rd trimester) and postnatal maternal depressive symptoms (at six months postpartum) among rural Bangladeshi women. In South Asia, women are often dependent on their husbands due to gaps in age, education, and control of financial resources [14]. Therefore, a second aim was to assess women’s reports of the couple’s discordance in intentions and unwantedness on the part of both parents in relation to women’s perinatal depressive symptoms.

## Methods

This study is a secondary analysis of data collected during the course of a cluster randomized, double-masked, placebo-controlled, community trial (JiViTA-1) of the effects of vitamin A or beta-carotene supplementation on all-cause, pregnancy-related mortality [15, 16]. The ClinicalTrials.gov identifier for that previous study was: NCT00198822, registered on 9/12/2005. JiVitA-1 was conducted between August 2001 and October 2007.

### Study setting

This trial took place in 19 rural unions of Gaibandha and Rangpur Districts in northwestern Bangladesh, comprising an area of ~ 435 km^2^. The site was selected to be typical of general living conditions in rural Bangladesh and eastern Gangetic region of South Asia. At the time of the study, about 40% of the married women of reproductive age in the area were literate, with < 5% having completed the 10th grade and < 40% were involved in income-generating work on their own. Husbands were mostly employed in subsistence agriculture, hired themselves out as daily wage laborers or ran a small business. Less than 15% of households had access to electricity, and households were mostly made of earth or tin.

### Pregnancy ascertainment

Married women of reproductive age (13–44 years) who were neither sterilized nor menopausal and living with their husbands were recruited and placed under pregnancy surveillance [14]. All women were visited every five weeks to ask about their menstrual history during the past 30 days and offered a urine test if amenstrual to identify incident pregnancies. After obtaining verbal consent (due to illiteracy among many participants), pregnant women were enrolled in the trial, visited for an enrollment interview and began receiving supplements as per their allocation.

### Data collection

Participants were interviewed by trained interviewers at inclusion (early pregnancy, median gestation age 9 weeks) to collect information on household socioeconomic factors (education, asset ownership, house construction), maternal nutritional status, diet, morbidity history, previous pregnancy history, wantedness of the incident pregnancy, hygiene, physical work history as well as tobacco and alcohol use [17]. Women were tracked through the course of their pregnancies and all women were interviewed again at 3 and 6 months post-partum and asked about labor and delivery, diet, morbidity history, work history and tobacco and alcohol use. In addition, from February 2004 to July 2017, a module of questions related to depression and suicide was introduced into the study and asked independently at two different times of eligibility, during the latter half of pregnancy (Median = 24.4 wks, Mean = 25.2 wks, SD = 2.1), and at ~ 6 months post-partum(Median 32.1 wks, Mean 32.4 wks, SD = 1.06 wks). All interviews were conducted one-on-one in women’s homes and sufficient community counseling ensured that interviews were private and confidentiality could be maintained. If a woman could not be interviewed alone at a specific time, the interviewer rescheduled the visit until it could be conducted privately.

Data were collected at enrollment about unintended pregnancy from the woman for herself (“Did you want to become pregnant now?”) and for her husband (“Did your husband want you to become pregnant now?”). Options included “wanted now”, “mistimed” or “unwanted”, asked approximately one week after a positive pregnancy test (Median = 1.0 wks; Mean = 11.6 wks, SD = 2.4). Pregnancy-intention discordance was defined from the mother’s report of her intentions and those of her husband. For the purpose of the couple’s discordance analysis, mistimed and unwanted pregnancies were combined and defined as “unwanted.” Using ‘wanted now’ and the collapsed category of “unwanted” pregnancy (for the purpose of the discordance analyses), couples’ discordance was defined as a non-match between the mother and her husband’s intentions (not either both “wanted” or both “unwanted”).

Because there was no validated depression scale in Bangladesh at the time of JiViTA-1, depressive symptom items were adapted from the nine-item Patient Health Questionnaire (PHQ-9) and the Center for Epidemiologic Studies Depression Scale (CES-D) [18, 19]. Selection of items (six prenatal and five postnatal) was based on pretesting in the study area. Questions were translated by a professional translator and an independent back-translation was done between Bangla and English to ensure that the meaning of the questions was retained. Women in the area struggled to grasp the meaning of some questions on the PHQ-9 and the CES-D, and so only questions that were clearly understood in focus group discussions were included. Two standard questions about suicidal ideation and suicide attempts were also included.

Maternal depressive symptoms during the latter half of pregnancy were measured using the following six symptoms reported in the last 30 days (yes/no): feeling sad all the time; becoming more forgetful; crying all the time; having thoughts of hurting yourself; sleeping more than before; and trying to hurt yourself. Postpartum maternal depressive symptoms were measured by asking women the first four items used in the prenatal period (listed above), along with a fifth symptom, not wanting to bathe or eat for several days in the last 60 days.

Socio-demographic and other covariates included: living standards index in quartiles (1st = low, 4th = high), maternal education (none, 1–9, ≥10 years), maternal literacy (yes/no), maternal age at enrollment (≤19, 20–29, ≥30 years), maternal nutritional status during pregnancy (mid-upper arm circumference in the third trimester: < 21.5; ≥21.5 cm), anemia in the first trimester (symptoms of breathlessness at rest resulting in inability to work from the World Health Organization maternal screening questions), and infection in the first trimester (either urinary tract infection or pneumonia or both). [20] The living standards index was created using Principal Components Analysis, combining several household asset variables (toilet facilities, type of walls, kitchen, and roof, the number of clocks, living rooms, closets, beds, radios, irrigation pumps, televisions, rickshaws, and having electricity) [21]. Participants were classified in vitamin A, beta-carotene, and placebo groups representing their micronutrient supplementation assignment in the original trial.

The study protocol was reviewed and approved by the Johns Hopkins Bloomberg School of Public Health Institutional Review Board (IRB, #H.22.01.01.11.A1) and the Bangladesh Medical Research Council (BMRC, BMRC/ERC/1998–2001/2405). The informed consent process included, when so desired by the subjects, husbands, in-laws and sometimes community members. Individual consent was sought from the participating women and documented.

### Data analysis

Descriptive demographic and pregnancy intention statistics were summarized for women in the subsample of the trial cohort who received prenatal and postnatal depressive symptoms assessments.

We restricted our analyses for prenatal depressive symptoms to women interviewed after the 23rd week and before the 37th week of gestation, in order to make sure that women were interviewed during a common time window, after a spontaneous loss (miscarriage) and before the very end of the pregnancy when hormonal changes related to pending delivery might affect their responses. Analyses were further restricted to women with singleton live births. The Cronbach alpha for prenatal symptoms with all items was 0.57 (unexplained variance = 0.67), and 0.63 (unexplained variance = 0.61) after removal of the item ‘sleeping more than before.’ The remaining five items were summed to achieve a total score of 0–5, with a score ≥ 3 considered our higher level of depressive symptoms. Only women with data on all five items were included in the analysis. Cronbach’s alpha was 0.71 (unexplained variance = 0.49) for the five postpartum depressive symptoms. Items were summed and a cutoff of ≥3 was our basis for dichotomizing depressive symptoms based on the fact that this was the cutoff that conservatively optimized the number of women with symptoms (8% prenatally, 13% postnatally) that would be expected in rural Bangladesh.

Bivariate risk ratios (RRs) and 95% confidence intervals (CI) were calculated using a generalized linear model with binomial error structure and logarithmic link function. Unintended pregnancy included mothers’ and their husbands’ intentions separately (wanted and mistimed versus unwanted) and discordance between intentions (comparing unwanted by the mother/perceived as unwanted by the husband; unwanted by the mother/perceived as wanted by the husband; wanted by the mother/perceived as unwanted by the husband; wanted by the mother/perceived as wanted by the husband). Adjusted RRs were calculated for intentions and depressive symptoms at both time points including potential confounders and covariates of theoretical significance. We did not adjust for prenatal depressive symptoms in models for postpartum depressive symptoms due to the fact that we consider prenatal depressive symptoms potentially on the causal pathway, e.g. the negative effects of unintended pregnancy might first cause depressive symptoms during pregnancy which are sustained after the birth.

All models adjusted for geographic clustering sampled in the original study design and mothers’ assigned vitamin supplementation group in the trial. All adjusted analyses at six months postpartum controlled for child sex. We were unable to include arm circumference, anemia and infection variables in all models simultaneously because of small numbers in some categories. Quasi-likelihood under the Independence Model Criterion Goodness of Fit tests indicated that arm circumference and anemia yielded the best fit; these two variables were included in our multivariable models.

## Results

**Fig. 1:**
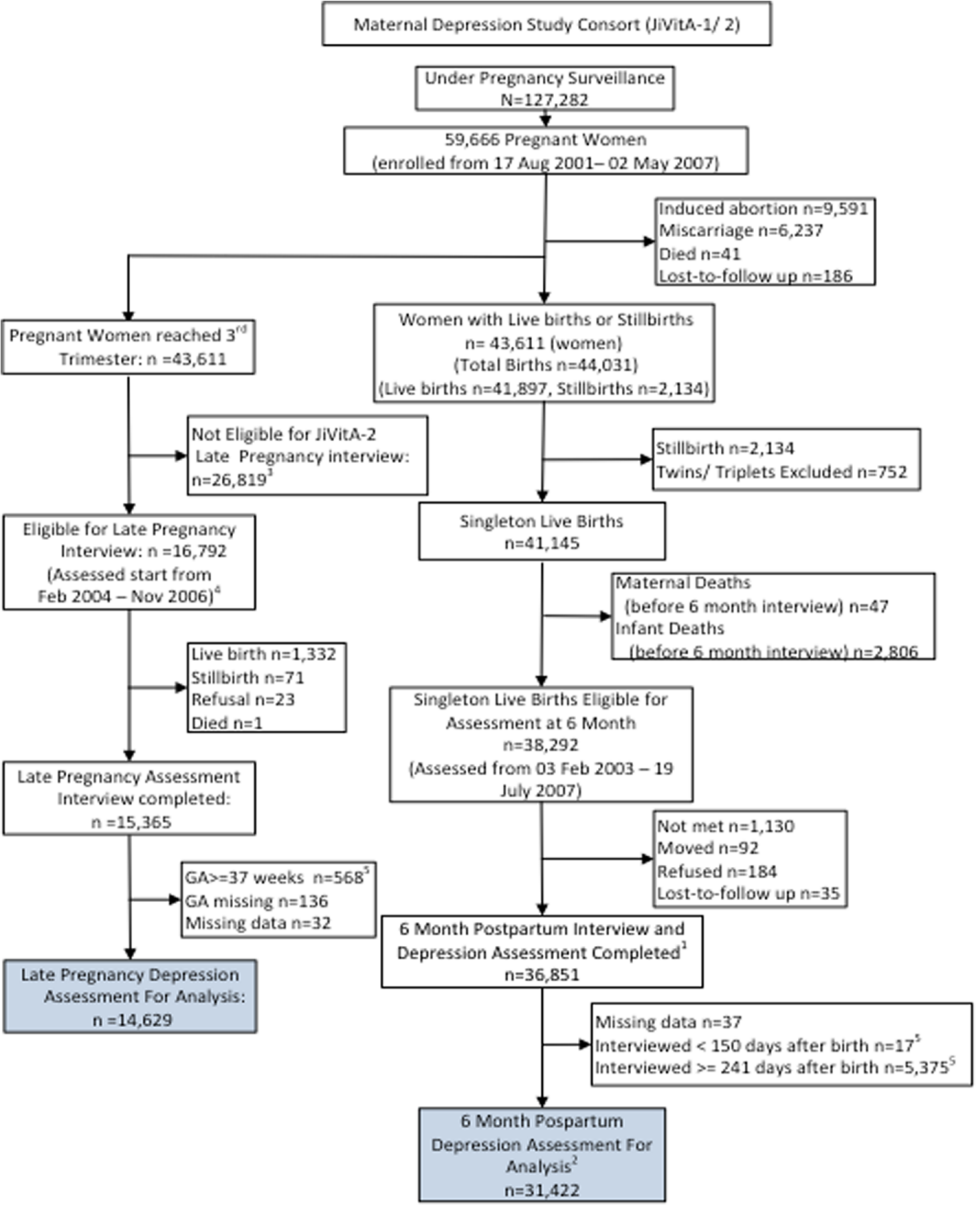
Flow diagram of study participants

A total of 127,282 women were placed under 5-weekly pregnancy surveillance, of whom 59,666 became pregnant, of whom 16,792 were in the subsample that was eligible for assessment of depressive symptoms while pregnant during the eligible period that, for pregnant women, ran from February 2004 through November 2006. Excluding pregnancies in this subset who delivered, refused or died being assessed and mothers not meeting the gestational age window of eligibility, we assessed 14,629 3rd trimester gravida for depressive symptoms at a median gestational age of 32.1 weeks (IQR = 31.9–32.7 wks; mean = 32.4 wks, SD = 1.06 wks) (Fig. 1). Among all 59,666 pregnant women, 38,292 delivered a live, singleton birth such that they were eligible for the 6 month post-partum depressive symptom assessment during the active period of this study protocol, from February 2004 through July 2007. Among these, 36,851 mothers were assessed postpartum; however, because this assessment occurred across a wider postpartum interval, this analysis has been restricted to mothers whose depressive symptom interview occurred between 150 to 240 days (5–8 months), inclusive, leaving 31,422 women in the present analytical cohort (Fig. 1).

For the subsample for which prenatal depressive symptoms were reported, 59, 31 and 11% of mothers reported having a wanted, mistimed and unwanted pregnancy, respectively (Table 1). The respective proportions for mothers’ perception of their husbands’ pregnancy intentions were 67, 24 and 10% for wanted, mistimed and unwanted pregnancies (Table 1). In the postpartum sample, 57, 31 and 12% of mothers reported having a wanted, mistimed and unwanted pregnancy. The respective proportions based on the mother’s perception of the fathers’ pregnancy intentions were 63, 25 and 12% for wanted, mistimed and unwanted pregnancies (Table 1).

**Table 1:** Crude risk ratios of maternal wantedness of pregnancy and perceptions as risk factors for maternal depressive symptoms

	Prenatal depressive symptoms *N* = 1201 (8.2%)		Postnatal depressive symptoms *N* = 3965 (12.6%)	
	Total (%)/Cases (%)	RR [95% CI]	Total (%)/ Cases(%)	RR [95% CI]
PREGNANCY INTENTIONS				
Maternal pregnancy wantedness				
Wanted now	7835 (58.5)/563 (6.7)	Reference	15,647 (57.1)/2015 (11.4)	Reference
Mistimed	4091 (30.5)/372 (8.3)	1.21* [1.07, 1.37]	8352 (30.5)/1110 (11.7)	1.02 [0.95, 1.10]
Not wanted	1474 (11.0)/266 (15.3)	2.32* [2.02, 2.66]	3393 (12.4)/834 (19.7)	1.73* [1.60, 1.87]
Perceived paternal pregnancy wantedness				
Wanted now	8868 (66.8)/701 (7.3)	Reference	17,218 (63.3)/2285 (11.7)	Reference
Mistimed	3129 (23.6)/261 (7.7)	1.04 [0.91, 1.20]	6875 (25.3)/872 (11.3)	0.97 [0.90, 1.04]
Not wanted	1272 (9.6)/223 (14.9)	2.06* [1.80, 2.37]	3118 (11.5)/768 (19.8)	1.70* [1.57, 1.83]
Parental discordance of pregnancy wantedness				
Unwanted by mother/perceived as unwanted by husband	3975 (30.0)/440 (10.0)	1.52* [1.34, 1.72]	9325 (34.3)/1527 (14.1)	1.26* [1.18, 1.34]
Unwanted by mother/perceived as wanted by husband	1497 (11.3)/186 (11.1)	1.62* [1.38, 1.90]	2285 (8.4)/396 (14.8)	1.26* [1.13, 1.39]
Wanted by mother/perceived as unwanted by husband	425 (3.2)/44 (9.4)	1.36* [1.01, 1.83]	666 (2.4)/113 (14.5)	1.23* [1.03, 1.48]
Wanted by mother/perceived as wanted by husband	7363 (55.5)/515 (6.5)	Reference	14,925 (54.9)/1889 (11.2)	Reference
DEMOGRAPHIC CHARACTERISTICS				
Maternal education				
None	5175 (38.6)/620 (10.7)	2.87* [2.17, 3.81]	12,447 (45.4)/2154 (14.8)	1.83* [1.59, 2.10]
1–9	7143 (53.2)/538 (7.0)	1.82* [1.37, 2.40]	12,844 (46.8)/1620 (11.2)	1.36* [1.18, 1.57]
≥ 10	1102 (8.2)/42 (3.7)	Reference	2136 (7.8)/187 (8.1)	Reference
Living standard index				
1st quartile (poor)	2439 (18.2)/294 (10.8)	1.73* [1.48, 2.02]	6672 (24.3)/1179 (15.0)	1.47* [1.35, 1.61]
2nd	3276 (24.4)/333 (9.2)	1.52* [1.31, 1.77]	6831 (24.9)/1045 (13.3)	1.32* [1.20, 1.44]
3rd	3689 (27.5)/307 (7.7)	1.24* [1.06, 1.45]	6917 (25.2)/940 (12.0)	1.18* [1.09, 1.29]
4th quartile (rich)	4023 (30.0)/267 (6.2)	Reference	7016 (25.6)/797 (10.2)	Reference
Maternal age (yrs)				
≤ 19	5742 (42.8)/430 (7.0)	Reference	11,522 (42.0)/1340 (10.4)	Reference
20–29	6332 (47.2)/536 (7.8)	1.15* [1.03, 1.30]	13,097 (47.7)/1926 (12.8)	1.25* [1.17, 1.33]
≥ 30	1343 (10.0)/235 (14.9)	2.22* [1.90, 2.59]	2821 (10.3)/699 (19.9)	1.93* [1.78, 2.10]
Previous births				
Zero	6252 (46.6)/437 (6.5)	Reference	11,209 (40.9)/1245 (10.0)	Reference
≥ 1	7176 (53.4)/764 (9.6)	1.51* [1.35, 1.69]	16,226 (59.1)/2717 (14.3)	1.44* [1.35, 1.53]
Maternal literacy				
Yes	6969 (51.9)/435 (5.9)	Reference	12,994 (47.4)/1487 (10.3)	Reference
No	6458 (48.1)/766 (10.6)	1.82* [1.63, 2.05]	14,442 (52.6)/2474 (14.6)	1.43* [1.34, 1.51]
Maternal employment				
Yes	3008 (22.4)/240 (7.4)	0.90 [0.79, 1.02]	4428 (16.1)/661 (13.0)	1.00 [0.92, 1.08]
No	10,419 (77.6)/961 (8.4)	Reference	23,008 (83.9)/3300 (12.5)	Reference
Religion				
Non-Muslim	1145 (8.5)/90 (7.3)	Reference	2219 (8.1)/281 (11.2)	Reference
Muslim	12,281 (91.5)/1111 (8.3)	1.13 [0.91, 1.42]	25,217 (91.9)/3680 (12.7)	1.17* [1.02, 1.35]
HEALTH-RELATED INDICES				
Mid-upper arm circumference (cm)				
< 21.5	2705 (20.2)/264 (8.9)	1.13* [1.00, 1.28]	6517 (23.9)/1057 (14.0)	1.16* [1.09, 1.23]
≥ 21.5	10,700 (79.8)/934 (8.0)	Reference	20,796 (76.1)/2884 (12.2)	Reference
Anemia during pregnancy				
Yes	4081 (30.4)/629 (13.3)	2.28* [2.04, 2.56]	8648 (31.6)/1769 (17.0)	1.61* [1.51, 1.70]
No	9341 (69.6)/571 (5.8)	Reference	18,697 (68.4)/2177 (10.4)	Reference
Infections				
None	12,069 (89.9)/979 (7.5)	Reference	24,844 (90.8)/3323 (11.8)	Reference
At least one or both	1354 (10.1)/221 (14.0)	1.79* [1.55, 2.06]	2505 (9.2)/624 (19.9)	1.61* [1.49, 1.74]
Vitamin supplementation				
Vitamin A (Z)	4386 (32.7)/424 (8.8)	1.07 [0.86, 1.34]	9144 (33.3)/1332 (12.7)	1.00 [0.86, 1.17]
Beta-Carotene (X)	4532 (33.8)/378 (7.7)	0.94 [0.76, 1.16]	9168 (33.4)/1306 (12.5)	0.98 [0.85, 1.13]
Placebo (Y)	4510 (33.6)/399 (8.1)	Reference	9145 (33.3)/1327 (12.7)	Reference
Child sex				
Male	–	–	13,839 (50.4)/2010 (12.7)	1.01 [0.96, 1.06]
Female	–	–	13,618 (49.6)/1955 (12.6)	Reference

*indicates *P* < 0.05

Analyses at six months postpartum are restricted to mothers who had singleton births. All models are adjusted for geographic sector

The total sample size for the *prenatal depressive symptoms* analysis was 14,629. The number of missing observations were: maternal pregnancy wantedness = 28; perceived paternal pregnancy wantedness = 175; perception of couple pregnancy discordance = 184; maternal education = 9; assets index, maternal literacy and maternal employment were missing one observation each; maternal age = 11; religion = 2; MUAC = 26; anemia = 7; infection = 6; None were missing from the parity or vitamin supplementation group

The total sample size for *postnatal depressive symptoms* analysis was 31,422. The number of missing observations were: maternal pregnancy wantedness = 71; perceived paternal pregnancy wantedness = 286; perception of couple pregnancy discordance = 296; maternal education = 34; assets index = 25; maternal age = 17; parity, maternal literacy, maternal employment, and religion were missing 25; MUAC = 168; anemia = 131; infection = 126; None were missing from the vitamin supplementation group or child sex

Finally, 8 and 13% of the mothers had ≥3 depressive symptoms during the prenatal and postnatal periods, respectively (Table 1). Mothers reporting a mistimed or unwanted pregnancy had a greater risk of prenatal depressive symptoms (RR 1.21, 95% CI 1.07, 1.37; RR 2.32, 95% CI 2.02, 2.66, respectively) than mothers with wanted pregnancies. Women reporting unwanted (RR 1.73, 95% CI 1.60, 1.87) but not mistimed pregnancies (RR 1.02, 95% CI 0.95, 1.10) were at greater risk for postnatal depressive symptoms (Table 1).

Women’s mistimed (Adjusted RR 1.26, 95%CI 1.11, 1.43) and unwanted pregnancies (Adjusted RR 1.60, 95%OR 1.37, 1.87) had higher risk of prenatal depressive symptoms than women with wanted pregnancies after socio-demographic adjustment. Postpartum depressive symptoms were not related to mistimed pregnancies (Adjusted RR 1.05, 95% CI 0.98, 1.13), but women with unwanted pregnancies had an elevated risk of postpartum symptoms (Adjusted RR 1.32, 95% CI 1.21, 1.44) (Table 2).

**Table 2:** Adjusted risk ratios of maternal wantedness of pregnancy and demographic as risk factors for maternal depressive symptoms

	Maternal Depressive Symptoms	
	Prenatal RR [95% CI] N = 14,554	Postnatal RR [95% CI] N = 31,178
Maternal wantedness of pregnancy		
Wanted now	Reference	Reference
Mistimed	1.26* [1.11, 1.43]	1.05 [0.98, 1.13]
Not wanted	1.60* [1.37, 1.87]	1.32* [1.21, 1.44]
Maternal education		
None	1.50* [1.05, 2.15]	1.29* [1.07, 1.54]
1–9	1.45* [1.07, 1.98]	1.29* [1.11, 1.50]
≥ 10	Reference	Reference
Living standard index		
1st quartile (poor)	1.19 [1.00, 1.43]	1.15* [1.04, 1.27]
2nd	1.13 [0.95, 1.33]	1.08 [0.98, 1.19]
3rd	1.03 [0.88, 1.22]	1.04 [0.95, 1.14]
4th quartile (rich)	Reference	Reference
Maternal age (yrs)		
≤ 19	Reference	Reference
20–29	0.94 [0.80, 1.11]	1.09* [1.02, 1.20]
≥ 30	1.38* [1.12, 1.70]	1.44* [1.29, 1.61]
Previous births		
Zero	Reference	Reference
≥ 1	1.00 [0.85, 1.18]	1.03 [0.95, 1.12]
Maternal literacy		
Yes	Reference	Reference
No	1.39* [1.15, 1.67]	1.13* [1.02, 1.25]
Religion		
Muslim	1.01 [0.81, 1.25]	1.09 [0.95, 1.25]
Non-Muslim	Reference	Reference
Prenatal mid-upper arm circumference (cm)		
< 21.5	1.00 [0.89, 1.13]	1.06 [0.99, 1.13]
≥ 21.5	Reference	Reference
Anemia		
Yes	2.08* [1.85, 2.33]	1.49* [1.40, 1.58]
No	Reference	Reference
Vitamin Supplementation		
Vitamin A	1.11 [0.90, 1.37]	1.00 [0.86, 1.16]
Beta-Carotene	0.97 [0.79, 1.18]	1.02 [0.89, 1.18]
Placebo	Reference	Reference
Sex of resulting birth		
Male	–	1.01 [0.96, 1.07]
Female	–	Reference

*indicates P < 0.05

Analyses during pregnancy include only women interviewed between at or after 23 weeks or before 37 weeks of gestation. Analyses at six months postpartum are restricted to mothers who had singleton births. All models are adjusted for geographic sector

Women with husbands who they perceived as not wanting the pregnancy were at higher risk for prenatal (Adjusted RR 1.42, 95% CI 1.22, 1.65) and postnatal depressive symptoms (Adjusted RR 1.30, 95% CI 1.19, 1.41), than women who perceived their husbands wanted the pregnancy (Table 3).

**Table 3:** Adjusted risk ratios of maternal perception of wantedness as risk factors for maternal depressive symptoms

	Maternal depressive symptoms	
	Prenatal RR [95% CI]	Postnatal RR [95% CI]
Paternal wantedness of pregnancy (*n* = 14,399; *n* = 30,964)		
Wanted now	Reference	Reference
Mistimed	1.10 [0.96, 1.26]	0.99 [0.92, 1.07]
Not wanted	1.42 [1.22, 1.65]*	1.30 [1.19, 1.41]*
Parental discordance of pregnancy wantedness (n = 14,399; n = 30,954)		
Unwanted by mother/perceived as unwanted by husband	1.34 [1.19, 1.52]*	1.13 [1.06, 1.21]*
Unwanted by mother/perceived as wanted by husband	1.51 [1.29, 1.78]*	1.24 [1.12, 1.37]*
Wanted by mother/perceived as unwanted by husband	1.30 [0.97, 1.74]	1.19 [0.99, 1.44]
Wanted by mother/perceived as wanted by husband	Reference	Reference

Analyses were conducted in the third trimester and at six months postpartum. All analyses were adjusted for maternal education (none, 1–9, ≥10 years); maternal age (≤19, 20–29, ≥ 30 years); parity (≥1, none); maternal literacy (no, yes); maternal employment (no, yes); religion (Muslim, non-Muslim); maternal mid-upper arm circumference in the first trimester (< 21.5, ≥21.5), anemia in first trimester (yes/no), vitamin supplementation group (vitamin A, beta-carotene, placebo) and geographic sector

Analyses for postnatal depressive symptoms were additionally adjusted for the sex of the child

In this table the “does not want” combines stating those who answered they did want the pregnancy and those who answered not wanting the pregnancy at this time

*indicates *P* < 0.05

Perception of mistimed pregnancy for husbands was not related to prenatal or postnatal depressive symptoms. Maternal report of not wanting the pregnancy and also perceiving her husband to not want the pregnancy was associated with a 34% higher risk of prenatal depressive symptoms (Adjusted RR 1.34, 95%CI 1.19, 1.52) compared to when both parents wanted the pregnancy. Similarly, having the mother not want the pregnancy but perceive her husband want it was associated with a 51% higher risk of prenatal depressive symptoms (Adjusted RR 1.51, 95% CI 1.29, 1.78), compared to cases in which both parents wanted the pregnancy. Finally, maternal report of wanting the pregnancy but perceiving that her husband did not want it was associated with a 30% higher risk of prenatal depressive symptoms (Adjusted RR 1.30, 95% CI 0.97, 1.74) compared to when both parents wanted the pregnancy (Table 3) compared to mothers who wanted the pregnancy and perceived that their husbands’ also wanted it. A similar, but less pronounced, increase in the risk of depressive symptoms associated with unintended pregnancy was observed in the postpartum period, i.e. a 13% increased risk for mothers who did not want the pregnancy and perceived their husbands did not want it (Adjusted RR 1.13, 95% CI 1.06, 1.21), a 24% increased risk for mothers who did not want the pregnancy but perceived their husbands wanted it (Adjusted RR 1.24, 95% CI 1.12, 1.37); and a 19% increased risk for mothers who wanted the pregnancy but perceived their husbands did not want it (Adjusted RR 1.19, 95% CI 0.99, 1.44).

Table 2 also shows multivariable results for the socio-demographic covariates and maternal depressive symptoms. Low maternal education was a risk factor for prenatal and postnatal depressive symptoms. Poor living standards showed an increased risk only for postnatal depressive symptoms. Older maternal age was associated with a higher risk of both prenatal and postnatal depressive symptoms. Anemia also was associated with greater risk of prenatal (Adjusted RR 2.08, 95% CI 1.85, 2.33) and postnatal (Adjusted RR 1.49, 95% CI 1.40, 1.58) symptoms. Religion, mid-upper arm circumference and child sex were not associated with depressive symptoms in any models (Table 2).

## Discussion

Our results suggest pregnancy intentions, especially unwanted pregnancies, are related to maternal depressive symptoms during and after the pregnancy among impoverished rural Bangladeshi women. The likelihood of maternal prenatal depressive symptoms was 60 and 26% higher among women reporting unwanted or mistimed pregnancies, respectively, after adjustment. During the postpartum period, having an unwanted pregnancy was associated with 32% higher risk of maternal depressive symptoms. Mothers’ perception that the pregnancy was unwanted by their husbands was also associated with a higher risk of prenatal and postnatal depressive symptoms. Finally, maternal perception of discordance between her own and her husband’s pregnancy intentions conferred a higher risk especially of prenatal depressive symptoms, with over a 50% higher estimated risk occurring when the mother did not want the pregnancy but perceived her husband did want it. This elevated risk remained at 6 months postpartum, but was attenuated.

Our results are consistent with existing literature for South Asia. In Pakistan, women with higher levels of depressive symptoms between 20 and 26 weeks of pregnancy were also more likely to report unwanted pregnancies [17]. In an Indian study, unplanned pregnancy was associated with depression in women at 6–8 weeks postpartum [22]. A meta-analysis, including five studies from lower- and middle-income countries, estimated 1.6 to 8.8 greater odds of maternal depressive symptoms associated with unwanted or unintended pregnancy [9]. In Bangladesh fathers’ intentions appear to have more influence on a birth occurring than mothers’ intentions [23]. Given these studies, it is possible that lack of control of fertility decisions in this context may contribute to women’s depressive symptoms associated with unwanted pregnancy.

We found pregnancies unwanted by the husband, as reported by the mother, were associated with subsequent maternal depressive symptoms, a finding consistent with studies conducted in the United States and Japan [24–26]. As poor quality spousal relationships and interpersonal violence are linked to depression in Bangladeshi women, it is possible that a husband not wanting a pregnancy may reflect less family involvement or a poor quality relationship with the mother and child [27]. We found that the scenario in which mothers reported not wanting the pregnancy but perceived their husbands wanted it was the kind of pregnancy discordance most highly associated with the risk of maternal prenatal depressive symptoms. It is possible that this reporting pattern, i.e. the mother’s perception that she did not want the pregnancy, but her husband did, may in some cases reflect sexual violence in which women are being forced to have sex in the context of their marital relationships.

We found an increased risk of prenatal and postnatal depressive symptoms for both discordant scenarios as well as for when the mother perceived that the child was unwanted by both parents, in comparison to when she perceived the pregnancy was desired by both husband and wife. To our knowledge only one study has examined this relation. In this US study of marital status and postpartum depressive symptoms, disagreement about pregnancy was significantly related to maternal depressive symptoms [28]. Perceived discordant pregnancy intentions may reflect conflict in the relationship or poorer relationship quality. Research from Bangladesh on pregnancy-intention discordance suggests fathers’ desires may carry more weight; women have a higher likelihood of a birth if the father desires the pregnancy and the mother does not but a lower likelihood of a birth if the mother, but not the father, desires the pregnancy [23]. Thus, it is possible that pregnancy-intention discordance may partially reflect circumstances in which woman’s desires are unfulfilled. When mothers did not want the pregnancy and perceived their husbands as not wanting the pregnancy may reflect circumstances in which the couple agreed that their condition was not ideal for having a child, either because of economic concerns or because they already had the number of children they desired.

The relation between unintended pregnancy and depressive symptoms can be viewed through a stressful life event lens [29]. Pregnancy and childbirth have been regarded as stressful life events and unintended pregnancy often exacerbates stress; for example, because of disruption of other life plans, being unprepared for parenthood, or lack of support [30]. Gender-based violence is pervasive in Bangladesh; over half of ever-married women age 15–49 report having experienced a form of physical or sexual violence from their husbands [31]. Of these women, 18% reporting his physically forcing her to have sex at some point, with 11% of those saying this occurred often or sometimes [31]. Spousal violence has been linked to suicidal ideation in Bangladesh and to poor mental health outcomes in India [32, 33]. Although we lacked data to examine the role of interpersonal violence, it is possible that sexual assault is a pathway to unintended pregnancy and is a root cause of the depressive symptoms we observed.

Maternal depressive symptoms and pregnancy intentions have been virtually unexplored among rural socio-economically disadvantaged women in developing countries, where unintended pregnancies are common and views toward childbearing and its cultural meaning may be different than in industrialized settings. The opportunity to follow a large cohort prior to pregnancy is rare in developing countries, with prospective data on pregnancy intentions gathered during the first trimester of pregnancy. Retrospective data on pregnancy intentions is thought to underestimate unwanted pregnancies. We also had data on perceived paternal pregnancy intentions, a topic with almost no published literature. The fact that mothers reported on their husbands’ pregnancy intentions is a limitation. However, mother’s perception of her husband’s attitude and perception of pregnancy-discordance between them may be, nevertheless particularly relevant to her mental health irrespective of his actual view. Another limitation was that there was no validated depression scale in Bangladesh at the time of our study, such that we used items drawn from several validated scales and suicide-related questions. As a result, we developed our own depressive symptom scales. The prenatal depressive symptom scale showed relatively low reliability based on Cronbach’s alpha. This low alpha value may have been due to having relatively few items in these scales or may reflect poor interrelatedness of these items. The generalizability of our study is limited to women who are married, carried their baby throughout pregnancy, and had singleton births.

## Conclusions

In summary, we found that maternal and paternal unwanted pregnancy, maternal report of mistimed pregnancy, and maternal report of couple pregnancy-intention discordance were risk factors for maternal depressive symptoms. Given that these symptoms may have important adverse consequences for mothers and children, knowledge that unwanted pregnancy is associated with an increased risk of depressive symptoms could be used to help identify women at risk. Especially in developing-country settings like rural Bangladesh where there is little funding and infrastructure for mental health, prevention efforts may take into account initiatives to prevent unwanted pregnancies and/or integrate family planning and mental health services. Future research is needed to understand the factors that determine whether pregnancies are unwanted or mistimed. Our results highlight an unmet need in Bangladesh, and likely other similar settings, for family planning services to target both wife and husband when offering pregnancy counseling to newly married couples in order to avoid unwanted pregnancies.
